# Gangrene Resulting from Bite of “Blue-Gum” Negro

**Published:** 1896-04

**Authors:** L. H. Ogbourn

**Affiliations:** Gallion, La.


### GANGRENE RESULTING FROM BITE OF "BLUE-GUM" NEGRO.

GALLION, LA., March 2, 1896.

*Atlanta Medical and Surgical Journal:*

With your permission I will report what is to my mind rather a peculiar case. A stout negro man, about thirty-five years old, came to my office with his left hand very much swollen, and upon careful examination I found the entire hand in a gangrenous condition. My first impulse was to amputate at the wrist, but after a moment's thought I concluded that I had better get the history of the case before operating, and upon inquiring I elicited the following history:

About ten days previous while in a fight with another negro the latter had gotten his fingers in his mouth, between the first and second joints, and bit it to the bone; not thinking that it would amount to anything, he had his wife to dress it with antiseptic, and for two or three days thought it was getting all right. About the fourth day it began to pain him very much, and he had his wife to redress it with an ointment of some kind, but it continued to grow worse, and becoming alarmed he applied to me; and as previously stated, I found the hand in a state of decomposition. But as the primary trouble had originated in the first finger I decided to practice conservative surgery and give him a chance for his hand. Therefore I proceeded with patient under the influence of chloroform to amputate the finger at the metacarpo-phalangeal articulation, and with my finger and handle of scalpel, I removed all unsound flesh from the entire hand, closed the wound with interrupted silk sutures, and dressed with iodoform gauze and absorbent cotton, and instructed patient to return on third day, which he did, and to my very great surprise I found nice, healthy granulations, and in due course of time he made a good recovery with the loss of one finger only.

Rather an unpleasant sequel is yet to come. Just before administering chloroform, I gave patient hypodermically $\frac{1}{4}$ gr. morphine sulph. with $\frac{1}{150}$ gr. atropine sulph. After getting through dressing the hand I waited the usual time for patient to revive, of course anticipating no trouble from that source, but as he did not respond as readily as I thought he should, I began bathing his face with cold water and he continued to sleep. After waiting a while longer, I again examined him and to my astonishment I found his pulse down to thirty and his respiration about five per minute. I at once gave him digitalin hypodermically, and whisky by the mouth, and after several hours he recovered.

Why should such a slight wound result so seriously? And what caused scuh profound coma after the administration of the anesthetic? Was it caused from the morphia or from the chloroform?

The anesthetizer was composed of a piece of heavy paper rolled into a cone shape and cotton put in bottom to hold the chloroform. The operation was performed in an open office with free circulation of air.

The patient said his antagonist had blue gums, and that was the reason why it made a poisoned wound. In his own vernacular "A blue gum nigger's bite is badder den a mad dog's; dey will sho pisen you every time." Yours truly,

L. H. OGBOURN, M.D.

